# Recurrence of Colorectal Cancer After Liver Transplant for Isolated Colorectal Liver Metastases: A Narrative Review

**DOI:** 10.3390/cancers18091450

**Published:** 2026-05-01

**Authors:** Rishav Agrawal, Anthony J. Choi

**Affiliations:** 1 Department of Medicine, Weill Cornell Medical College, New York, NY 10021, USA; rishava98@gmail.com; 2 Department of Gastroenterology and Hepatology, Weill Cornell Medical College, New York, NY 10021, USA

**Keywords:** colorectal cancer, liver transplant, recurrence, colorectal liver metastases

## Abstract

Liver transplant can significantly improve survival in patients with isolated colorectal liver metastases (CRLMs) not amenable to surgical resection. The use of evolving strict selection criteria to identify patients with CRLM best suited for transplant has greatly prolonged disease-free survival and resulted in a post-transplant overall survival similar to that of other established indications for transplant such as advanced cirrhosis or hepatocellular carcinoma. While recurrence of colorectal cancer is still seen in the majority of patients after transplant, lesions are typically slow-growing and treatable without significant mortality. Further profiling of patients’ tumor molecular and biologic activity before transplant can help risk-stratify them for aggressive recurrence. Our current understanding of recurrence after liver transplant for CRLM is based on only a small number of prospective studies but further trials are underway. Novel surgical approaches which could expand the pool of patients eligible for transplant are also being evaluated. We summarize and discuss the current state of the literature describing the patterns, risk factors, and treatment of recurrence after liver transplant for isolated CRLM.

## Simple Summary

The liver is the most common site for metastases of colorectal cancer, and many patients are left with cancer isolated in the liver even after surgical resection of their primary tumor. Liver transplantation is a promising treatment option for these patients who are otherwise not eligible for further therapies and can drastically improve their long-term survival. Historically, it was common to have recurrence of cancer after transplant, most often in the lungs, but these recurrences are usually slow-growing and amenable to treatment and even cure. Recent advances in selection criteria for determining which patients should undergo transplantation have also resulted in lower recurrence rates and a higher long-term overall survival similar to what has been seen for other common indications for liver transplant. In this narrative review, we summarize how the incidence and patterns of recurrence have changed over time. We also detail our current understanding of the risk factors and treatment options for recurrent disease after liver transplant for isolated colorectal liver metastases.

## 1. Introduction

Colorectal cancer (CRC) is the third most diagnosed cancer in the United States and the second most common cause for cancer death [1]. The liver is the most common site of CRC metastasis, with an estimated 17% of patients with CRC having synchronous liver metastases at diagnosis and an additional 13% of patients later diagnosed with metachronous liver metastases. Among those with colorectal cancer liver metastases (CRLMs), about two-thirds have isolated CRLM without evidence of extrahepatic metastases [2]. Historically, surgical resection was the only curative treatment option for these patients. However, a majority of patients have unresectable disease due to various factors such as the extent of metastases, involvement of unresectable structures, or the volume of functional liver involvement, resulting in an estimated overall survival of less than 20 months prior to the routine use of liver transplantation for CRLM [3,4,5].

Within the growing field of transplant oncology, liver transplantation (LT) is widely used for the treatment of primary hepatocellular carcinoma (HCC) with 5-year survival rates greater than 70% [6]. There has long been interest in the use of LT for isolated CRLM, though attempts in the 1990s resulted in poor outcomes with 5-year overall survival measured at less than 20% [7]. In 2013, with advances in pre-transplant imaging modalities for proper staging, transplant surgical techniques, and post-transplant immunosuppression, a pilot study in Norway demonstrated a significantly improved 5-year survival of 60% [8]. Despite significant improvements in overall survival, it has been consistently shown that LT for CRLM is rarely curative and is associated with a high rate of recurrence [8]. This surprising contrast between a high overall survival but low recurrence-free survival is unique to LT for CRLM and has not been seen in LT for primary HCC, which can be associated with a recurrence rate of less than 15% when patients are within Milan criteria [9,10,11].

We seek to summarize the current state of the evidence describing the incidence, patterns, and treatment options of recurrence of CRC after LT for isolated CRLM.

## 2. Methods

We conducted a structured literature search to achieve a broad overview of available English-language studies discussing LT for CRLM. The Mesh terms “Colorectal Neoplasms” and “Liver Transplantation” were queried on PubMed as well as the general search terms “colorectal liver metastasis”, “CRLM”, “colorectal cancer”, “transplant”, and “recurrence” on PubMed and Google Scholar. Results were filtered to articles, equivalence or clinical trials, evaluation, observational or validation studies, meta-analyses, practice guidelines, and reviews. Case reports and standalone abstracts were not included. Titles and abstracts were reviewed for relevance and the full text of relevant manuscripts were critically reviewed prior to inclusion. Given the scope of this narrative review and the inherent risk of bias in the few existing studies on the topic, we did not formally assess for the risk of bias in the included studies.

## 3. Prospective Studies

The current landscape of LT for CRLM is based on a small number of prospective studies beginning with the pilot SECA-I study published in 2013. Current guidelines [12] and much of the existing literature are based around the findings of these studies and further retrospective analyses of their cohorts.

The SECA-I study was a landmark prospective pilot study conducted in Norway between 2006 and 2011 which re-established LT as a treatment option for unresectable isolated CRLM. It included 21 patients with CRLM, no extrahepatic disease, and complete radical excision of their primary tumor. Inclusion criteria were otherwise broad, and patients were required only to have no standard contraindications to liver transplant, a good performance status (ECOG 0–1), and to have completed at least 6 weeks of systemic chemotherapy. There was notably no exclusion criteria related to tumor size, biology, or response to chemotherapy, and 16 of the 21 patients had progressive disease at the time of inclusion despite systemic chemotherapy. No adjuvant chemotherapy was provided after transplant. While this study demonstrated an estimated 5-year overall survival of 60%, its cohort also experienced a 100% recurrence rate for patients observed beyond 11 months and median time to recurrence of only 8 months after transplant. The authors suggested this high rate of recurrence could be related to immunosuppressive therapy post-transplant and attributed the conversely high overall survival to these recurrences being slow-growing and accessible to local excision or radiation. They also identified several risk factors to guide stricter inclusion criteria for further studies [8].

Building on the findings of SECA-I, the SECA-II study was conducted in the same center with 15 patients who underwent LT between 2012 and 2016 with more strict inclusion criteria. In addition to the criteria of SECA-I, patients were required to be at least one year from CRC diagnosis and have demonstrated a positive response to pre-transplant systemic chemotherapy without progressive disease. It found an improved recurrence rate of 53% and significantly improved 5-year OS of 83%. These findings highlighted the effect of careful patient selection on lower recurrence rates and a high OS similar to that of other accepted indications for transplant [13].

Given shortages of deceased-donor liver allografts outside of Norway, where SECA-I and SECA-II were conducted, there has been growing interest in the use of living-donor liver transplants (LDLT) for CRLM. Hernandez-Alejandro et al. published a prospective cohort study in 2022 describing 10 patients who underwent LDLT for isolated CRLM at three centers in North America between 2017 and 2020. It employed a similarly strict inclusion criteria to SECA-II and demonstrated comparable disease-free and overall survival, suggesting that LDLT was a viable alternative to deceased-donor liver transplant (DDLT), especially in regions with low decreased donor availability [14].

Another alternative to LDLT that has been explored is the use of extended criteria donor allografts, which do not meet standard criteria due to factors such as advanced age, donation after circulatory death, or prior malignancy or hepatitis B infection [15]. The SECA-II arm D study included 10 patients who did not meet the inclusion criteria for the primary SECA-II study due to the presence of extrahepatic disease or less than a 10% response to systemic chemotherapy pre-transplant. Nine of these patients received an extended-criteria donor allograft while one received a standard-criteria organ. These patients were observed to have a much higher recurrence rate, worse overall survival, and worse survival after recurrence compared to those in SECA-II. These worse outcomes were driven by more frequent and aggressive recurrence rather than allograft dysfunction, further supporting the strong relationship between pre-transplant disease tumor biology and post-transplant recurrence and death [16].

Finally, TransMet, the most recent large prospective study and only randomized controlled trial of LT for CRLM, compared neoadjuvant chemotherapy and DDLT to chemotherapy alone for the treatment of isolated CRLM across 30 centers in Europe. It compared 36 patients who underwent DDLT to 38 similar patients receiving chemotherapy alone with less strict inclusion criteria than other recent studies, only requiring three months of response to systemic chemotherapy compared to the one year recommended by current guidelines [12]. The authors found a comparable overall survival and recurrence rate to SECA-II and recent North American studies and demonstrated an enormous survival advantage for liver transplantation when compared to chemotherapy alone. The liver transplant cohort experienced an estimated five-year overall survival of 73% and median time to recurrence of over 17 months, both orders of magnitude higher than the 9% median five-year survival and 6.4-month progression-free survival seen in the comparison chemotherapy-only group. These findings helped establish LT as a standard option for the treatment of isolated, unresectable CRLM in select patients [17].

## 4. Incidence of Recurrence

The incidence of recurrence after LT for CRLM varies greatly among existing studies, which are heterogenous in their inclusion criteria and duration of follow-up (Table 1). The highest observed recurrence rate in the contemporary literature was the 90% seen in SECA-I, which had a median follow-up of 27 months. Most patients had early recurrence with a one-year disease-free survival (DFS) of only 35% [8]. Patients in the SECA-II study had a longer median follow-up of three years yet a lower recurrence rate of 53%, though most patients with recurrence still had early relapse within one year as demonstrated by the one-year DFS of 53% [13]. The SECA-II arm D study, which lacked strict inclusion criteria, found a high recurrence rate of 80% and correspondingly low DFS of 30%, both similar to that of SECA-I [16].

More recent studies of LT for CRLM, predominantly of LDLT in North America, have found remarkably low recurrence rates and higher one-year DFS when compared to the landmark trials out of Norway, though with much shorter median follow-up. Hernandez-Alejandro et al. examined 10 patients undergoing LDLT in North America and found an overall recurrence rate of just 30% and a one-year DFS of 62%, both substantially improved from SECA-II. An interim study of seven patients who underwent LDLT at one center in Canada, some of whom may have been included in Hernandez-Alejandro at al., had a similarly low recurrence rate of 29% and an even higher one-year DFS of 86%. Another retrospective study of 20 patients who underwent LT at a single center in the USA found an even higher estimated one-year DFS of 90%, though did not report median follow-up or the overall frequency of recurrence [20]. A larger retrospective study using the United Network Organ Sharing database examined 46 patients who underwent LT for CRLM and found a low overall recurrence rate of 22% and one-year DFS of 75%, albeit with an even shorter median follow-up of only 12 months. This large retrospective study estimated a three-year DFS of 53.7%, more consistent with what was seen in SECA-II [19]. Another large and more recent study in NA examined 26 patients who underwent LT at two centers and found a recurrence rate of just 33% with a median follow-up of 21 months [21]. The reductions in early recurrence observed in these studies may be related to differences in patient selection, more aggressive pre-transplant treatment strategies, and/or potential advantages of LDLT. Much of the large reduction in gross recurrence rate, though, is likely driven by a shorter median follow-up than the large European studies.

The largest prospective trial of LT for CRLM, TransMet, found a high one-year DFS of 83% among 38 transplanted patients across Europe, consistent what has been seen in contemporaneous North American studies. It had the longest median follow-up of all major studies in the field at 59 months and a correspondingly high overall recurrence rate of 72%, further suggesting that modern selection criteria have been effective at delaying the onset of recurrence and reducing its mortality [17].

## 5. Patterns of Recurrence

**Figure 1:**
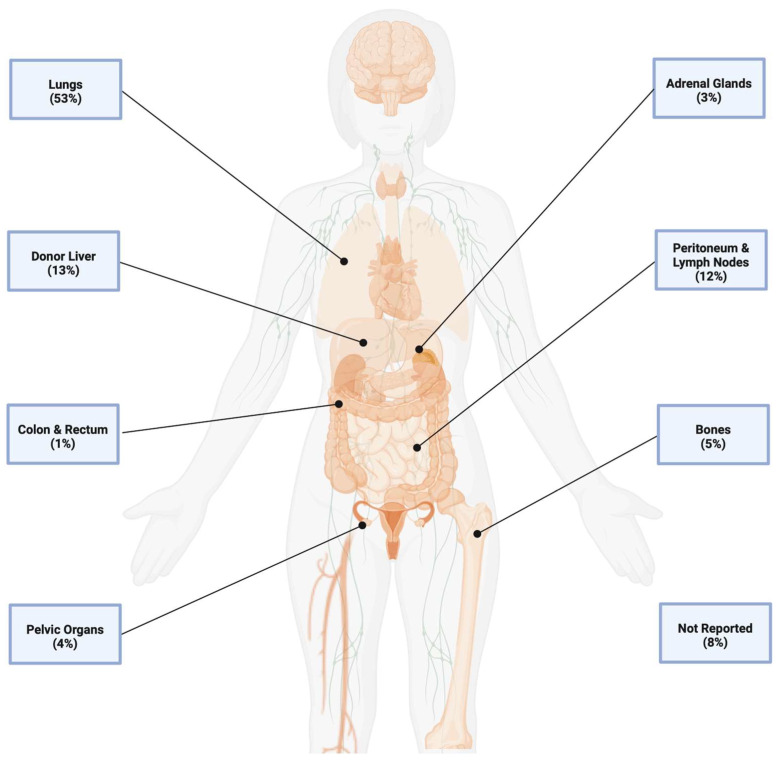
Sites of recurrence after liver transplant for isolated colorectal liver metastases. Visualization of the various sites of recurrence noted in the surveyed studies. Percentages represent the proportion of each site relative to the total number of sites reported. For patients with multiple sites of recurrence, some studies presented each site separately while others simply presented them as “multiple”. These patients are grouped together as not reported with the sites reported as “other”. Created in BioRender. Agrawal, R. (2026) https://BioRender.com/ha63vzt (accessed on 28 April 2026).

The sites of recurrence reported in the surveyed studies are presented in Figure 1. Most recurrences after LT for CRLM are solitary pulmonary metastases, similar to what has been observed after LT for HCC, though at a much higher frequency [22]. Every patient in the cohort of SECA-I had a pulmonary recurrence, though these were observed to be predominantly slow-growing nodules which were amenable to surgical resection and did not significantly impact mortality [23]. Pulmonary recurrences are generally found on surveillance CT scans of the chest, with a median pulmonary nodule size of 7.5 mm in diameter as reported in SECA-II [13]. Isolated pulmonary recurrences were found at a median of just four months after transplant in SECA-I [8], though this has improved significantly in recent studies with a median disease-free survival of over 13 months in SECA-II [13] and 17 months in TransMet [17]. Modern selection criteria for LT emphasizing a positive response to systemic chemotherapy and period without observed progression before transplant likely selects for patients with less aggressive and very-slow-growing metastases, explaining at least in part this significant improvement in DFS.

In the SECA I cohort, recurrence in the transplanted liver was generally seen as part of disseminated disease and all patients with liver recurrence had poor outcomes and high mortality. This was noted to be in contrast to liver recurrence after HCC, which is often isolated and associated with similar outcomes to isolated pulmonary recurrences [24]. Later studies, including TransMet and North American LDLT cohorts, had a small number of patients with isolated hepatic recurrences with better outcomes and post-recurrence survival, still generally inferior to that seen with isolated pulmonary recurrences but more in line with what is seen in liver recurrences after transplant for HCC [13,14,17]. It has been hypothesized that liver recurrences after LT for HCC may related to pre-existing malignant cells in the portal vasculature [25], which could also help explain this more aggressive early recurrence after LT for CRLM. The most common other sites of recurrence reported include the abdominal lymph nodes, peritoneum, pelvic organs, and bones. In general, these other extrapulmonary metastases are also associated with a more rapid progression and a high mortality if not amenable to surgical resection [24].

As with LT for HCC, recurrence after transplant is likely secondary to undetected malignant disease present at the time of transplant, and there are several reports of patients with recurrence that were found on retrospective review to have had missed evidence of extrahepatic disease in their pre-transplant workup. Seven of the 13 patients with early solitary pulmonary recurrence in SECA-I and one patients who underwent LDLT in a North American study were found to have small pulmonary nodules on pre-transplant CT scans that corresponded to the areas of recurrence [18,24]. A patient with early recurrence in the peritoneum after LDLT in Hernandez-Alejandro et al. was found to have evidence of metastatic disease in portal lymph nodes in their hepatectomy specimen which was not visible on pre-transplant CT or PET imaging [14]. Further advances in pre-transplant imaging modalities and protocols could reduce the incidence of recurrence after LT, which is in reality progression of persistent disease.

## 6. Risk Factors for Recurrence

Many pre-transplant prognostic factors have been identified to help determine which patients are at the highest risk for progression and mortality after LT for CRLM, including different risk scores and baseline tumor and patient characteristics.

### 6.1. Fong Clinical Risk Score

The Fong Clinical Risk Score (FCRS) was initially developed to predict recurrence after surgical resection of CRLM but has also been evaluated for use in risk stratification prior to LT for CRLM. The FCRS examines five criteria and suggested that those with more than two were at high risk for poor outcomes: node-positive primary tumor, <12 month disease-free interval from primary tumor detection to metastasis, multiple hepatic tumors, a largest hepatic tumor > 5 cm in diameter, and a carcinoembryonic antigen (CEA) level > 200 ng/mL (Table 2) [26]. In a 2023 retrospective cohort study of 61 patients included in SECA-I, SECA-II (including arm D), and the currently recruiting SECA-III and RAPID studies, a FCRS of <3 was associated with a median DFS of over 23 months, over double the DFS of 9.7 months observed in those with a FCRS of 3–5 [27].

### 6.2. Oslo Score

Based on the risk factors most associated with a poor outcome in SECA-I, the authors of the SECA-II study proposed the Oslo Score, a dedicated risk stratification tool for LT in CRLM. The OS includes four pre-transplant characteristics associated with poor prognosis after LT: a largest hepatic tumor > 5.5 cm in diameter, plasma CEA level > 80 mg/L, interval between primary CRC tumor resection and LT of <2 years, and progressive disease on chemotherapy at the time of LT (Table 3) [13]. In the SECA cohorts, an Oslo Score of 0–2 was associated with a DFS of 13.3 months, compared to only 6.1 months in those with a score of 3–4 [27].

### 6.3. Metabolic Tumor Volume

Both the FCRS and Oslo score consider the anatomic size of the largest hepatic tumor pre-transplant. This approach does not differentiate between metabolically active malignant tissue and necrotic tissue left behind after neoadjuvant systemic or locoregional therapy. Measurement of the metabolic tumor volume (MTV) on pre-transplant ^18^F -FDG PET/CT can be used to estimate the extent of a tumor which is metabolically active to provide a better measure of tumor aggressiveness before transplant. A 2022 retrospective study of 40 patients that included SECA-I and SECA-II compared patients with a low (<70 cm^3^) versus high (>70 cm^3^) MTV and found that patients with a high MTV had a recurrence rate of 100% and a median DFS of 4 months compared to a recurrence rate of 73% and DFS of 16 months in those with a low MTV [28]. This MTV cutoff was validated in a 2025 study of 26 patients who underwent (predominantly living-donor) LT at two centers in the United States and found that an MTV < 70 cm^3^ was associated with a median recurrence-free survival of 4.1 years as compared to 0.83 years in those with an MTV > 70 cm^3^ [21]. Current international consensus guidelines recommend that pre-operative MTV should be combined with a patient’s response to systemic therapy to reflect the biological activity of a tumor during transplant evaluation [12].

### 6.4. Circulating Tumor DNA

Laboratory testing for circulating tumor DNA (ctDNA) is another promising avenue for risk-stratifying patients for recurrence after transplant, though data supporting its use remains limited. A retrospective single-center study in 2023 examined four patients with positive pre-transplant ctDNA but negative post-transplant ctDNA testing and found that none had recurrence with a mean follow-up of 271 days. Conversely, five patients in this study had a positive post-operative ctDNA, three (60%) of whom had an observed recurrence. The authors were unable to assess for any association between a positive post-operative ctDNA and CRC recurrence among LT patients alone but found a positive association when also including patients who underwent resection of CRLMs [29]. Another study examining 18 patients who underwent LT for either HCC, cholangiocarcinoma, or CRLM and had post-operative ctDNA testing found a trend of higher recurrence that did not reach significance among those with a positive ctDNA [30]. Current international consensus guidelines state that ctDNA testing may be useful but less so than assessing patients’ response to neoadjuvant systemic chemotherapy before transplant evaluation [12]. Further research is required to understand the utility of ctDNA testing to predict recurrence after LT for CRLM.

### 6.5. Tumor Mutations

Several tumor genotypes are associated with more aggressive variants of CRC and may affect the risk of recurrence after LT. The best understood molecular marker in CRC is the BRAF V600E mutation, which is found in approximately 1–6% of patients undergoing resection for CRLM and has been associated with an over 50% decrease in overall survival when compared to BRAF wild-type patients in multiple large cohort studies [31,32]. Given the aggressive phenotype this mutation portends, patients with BRAF V600E-mutated CRC rarely have isolated CRLM (as opposed to more widely metastatic disease) and it is considered an absolute contraindication to liver transplant in current international consensus guidelines [12]. Patients with BRAF mutations have generally been excluded from studies of LT in CRLM, though the SECA-II arm D study included two patients with BRAF mutations. One of these patients had recurrence three months after transplant and an overall survival of only six months, while the other was alive and without recurrence at the end of 26 month follow-up [16]. One patient with a BRAF D594G mutation underwent LDLT in a North American study with a good outcome, though this variant is thought to confer a tumor phenotype more similar to the wild type than the aggressive V600E mutant [14].

Mutations in the KRAS gene have also been found in an estimated 30% of patients of with CRLM undergoing resection and been associated with a two-fold increase in recurrence [33]. Unlike BRAF V600E, current guidelines do not exclude patients with RAS mutations from LT as they have been less studied and are less frequently used to prognosticate CRC in routine clinical practice [12]. In a retrospective analysis of 19 patients from SECA-I and SECA-II, five patients had KRAS mutations and were found to have a similar overall survival to KRAS wild-type patients [34]. The SECA-II arm D study [16] and Hernandez-Alejandro et al. [14] each included three patients with KRAS mutations in their cohorts but did not specifically comment on their outcomes.

The effects of other genetic markers, including PIK3CA, PTEN [35], P53 [36], and SMAD [37] is an area of further research for which little data exists in regard to prognostic value in LT for CRLM. Owing to their favorable response to immunotherapy with generally good outcomes, patients with DNA mismatch repair or high microsatellite instability are not recommended to undergo LT and their outcomes after LT for CRLM have also not been well studied [12].

### 6.6. Primary Tumor Location

It is well understood that patients with primary CRC in the proximal colon have poorer outcomes than those with primary tumors in left colon. This is likely related to later discovery of more proximal disease leading to more advanced malignancy at diagnosis and to a generally worse response to systemic chemotherapy, among other factors [38]. Most patients in existing studies of LT for CRLM had left-sided colonic disease with very few having a right-sided tumor before resection and transplant. In a retrospective analysis of patients from the SECA group, patients with right-sided primary CRC tumors had earlier recurrence, with a median DFS of 3.3 months as compared to 9.0 months in those with left-sided tumors, and drastically worse survival after recurrence, 5.7 months versus 59.4 months in those with left-sided disease [39]. The remaining large cohort studies of LT for CRLM, including TransMet [17] and recent North American studies [14,18,21] did not compare recurrence by primary tumor anatomical location.

### 6.7. Post-Transplant Immunosuppression

No existing studies have directly compared different post-transplant immunosuppressive regimens after LT for CRLM. Mechanistic target of rapamycin (mTOR) inhibitors have been found to reduce the risk of de novo cancers after both LT [40] and renal transplant [41]. Studies of patients after LT for HCC have also found that the use of mTOR inhibitors rather than calcineurin inhibitors for maintenance post-transplant immunosuppression is associated with a higher overall and recurrence-free survival, particularly within the Milan criteria [42]. This may be related to suppression of anti-tumor immune activity by calcineurin inhibitors as well as possible promotion of tumor growth factors [43]. mTOR inhibitors may improve post-transplant recurrence-free survival both by inhibiting pathways involved in tumor growth and by minimizing calcineurin inhibitor use [44]. Based on these experiences, current international guidelines also recommend minimizing the use of calcineurin inhibitors for maintenance immunosuppression after LT for CRLM and instead favor the use of mTOR inhibitors either alone or in combination with a low-dose calcineurin inhibitor [12]. Patients in the SECA cohorts received sirolimus and mycophenolate mofetil maintenance therapy after completing induction immunosuppression [8,13], while those in TransMet [17] and North American LDLT trials [14] received either sirolimus or everolimus monotherapy for maintenance.

Pursuing more tailored immunosuppressive regimens based on pre-transplant tumor biology is a potential avenue for further research that has not yet been explored in LT for CRLM. It is possible that patients with more aggressive pre-transplant tumor activity, for example, may benefit from more rapid tapering of steroids and calcineurin inhibitors used for induction immunosuppression and earlier initiation of maintenance mTOR inhibitors. Conversely, those with less aggressive pre-transplant tumor biology could benefit from prioritizing graft preservation with increased or more prolonged use of calcineurin inhibitors given a theoretical lower risk of recurrence.

## 7. Treatment and Outcomes After Recurrence

The treatment of recurrence after LT for CRLM is dependent on location and includes the use of targeted surgical resection, locoregional therapy, and systemic chemotherapy. Immune therapy is generally avoided due to carrying increased risk for graft rejection, which has been seen in over 25% of patients treated with immune checkpoint inhibitors after LT for HCC [6].

The most common recurrences after LT for both HCC and CRLM are isolated pulmonary metastases, which are typically treated with local resection or ablation with good outcomes [25]. In SECA-I, all patients with isolated pulmonary recurrences were alive at the end of follow-up, with two undergoing resection without any residual disease and another with some remnant pulmonary metastases after resection. The remaining patients were awaiting resection or undergoing surveillance of pulmonary lesions not amenable to resection, typically due to small size. Among patients with pulmonary recurrence who then developed additional recurrence at extrapulmonary sites outside of the liver (including the ovaries, colon, and ribs), all underwent additional resections and were alive at the end of the study’s follow-up period. In contrast, all three patients with initial pulmonary recurrences which then spread to the liver died during the study, including one patient with a liver lesion amenable to resection. This resulted in an overall 5-year survival of 72% among patients with first-site pulmonary recurrence [24]. Five patients in SECA-II also underwent resections of pulmonary metastases with a median time from relapse to resection of 21.4 months, four of whom had no evidence of disease at the end of follow-up [34]. In TransMet, most recurrences observed were restricted to the lungs and 46% of recurrences were treated with surgery or local ablation [17]. In 2024, one patient in North America underwent successful double lung transplantation for unresectable isolated lung recurrences after LDLT. They had no evidence of residual disease on hospital discharge, though the long-term outcomes after lung transplant for this indication remain unknown [45,46].

Isolated recurrence outside of the lungs is also typically treated with local resection whenever possible and generally has good outcomes. Patients in SECA-I had local resections of initial recurrences in the rectum, ovaries, and lymph nodes, all with good outcomes and survival at the end of follow-up [24]. In SECA-II, one patient had local resection of a solitary liver metastasis and had no evidence of disease at the end of follow-up. Another two patients received surgical resection and local radiation for primary lymph node recurrences, though their specific outcomes were not reported [13]. In TransMet, local resection of metastases (lung and otherwise) led to secondary remission in 25% of patients and a secondary five-year DFS of 36%, suggesting that cure may be possible even after recurrence in some patients receiving LT for CRLM [17]. Localized radiofrequency ablation is another theoretical treatment option for isolated recurrences and has been used to treat unresectable CRLM in patients not undergoing LT [47], though this strategy has not been specifically reported for treating recurrence after LT.

Patients found to have more disseminated metastatic disease or local recurrence not amenable to surgical resection after LT are generally treated with palliative chemotherapy. In SECA-I, disseminated disease always involved the liver and resulted in very poor outcomes despite treatment with palliative chemotherapy and, in one case, trans-arterial chemoembolization of liver metastases [24]. A retrospective analysis of SECA I, SECA-II, and SECA-II arm D found a median OS after starting palliative chemotherapy of 13.1, 17.4, and 8.6 months, respectively, with an estimated 70% of patients having clinical benefit from first-line chemotherapy. A variety of chemotherapy regimens were employed, including capecitabine or irinotecan monotherapy as well as combination regimens such as Nordic FLIRI or FLOX with or without bevacizumab anti-EGFR therapy. The median duration of chemotherapy treatment was 11 months and it was generally well-tolerated without any observed cases of graft rejection, even as patients’ mycophenolate mofetil was stopped (with continuation of sirolimus monotherapy) with the start of chemotherapy [48]. In recent North American studies, a small number of patients with recurrence were treated with palliative chemotherapy, though short median follow-up precluded assessment of long-term outcomes [14,18].

## 8. Future Directions

Many proposed and in-process studies seek to further increase our understanding of recurrence and other outcomes after LT for CRLM. These include trials evaluating outcomes with even more strict inclusion criteria [49], living donors [50], as well as further randomized controlled trials comparing outcomes between LT and the otherwise best available treatments [51,52]. There are also novel surgical approaches under consideration which could expand the available donor pool, including evaluation of the RAPID (resection and partial liver segment 2–3 transplantation with delayed total hepatectomy) procedure [53] and a combination of two-stage hepatectomy and LDLT [54]. These studies, as well as further prospective and retrospective reviews of patients undergoing LT for CRLM in routine clinical practice, will help further detail the patterns, risk factors, and treatment options for recurrence.

Data becoming available in the HCC recurrence literature may also guide further research into recurrence after LT for CRLM. This includes exploring the effects of cold and warm ischemia time, as current retrospective data in HCC suggests that reduced ischemia time may be associated with a lower risk of recurrence [55]. This is increasingly relevant in LT for CRLM with the increasing use of LDLT and machine perfusion for deceased-donor livers, both of which may reduce ischemia time before transplant. Recurrence outcomes between LDLT and DDLT more generally also remain an important area for future exploration, as retrospective data in HCC suggests that LDLT may actually be associated with an increased risk of recurrence [56]. This is in contrast to the positive outcomes seen in recent studies of LDLT for CRLM, and further comparative analyses are needed to help distinguish the benefits of living-donor transplantation itself from that of the stricter inclusion criteria of these more recent studies.

## 9. Conclusions

Liver transplantation has emerged as a revolutionary treatment option for patients with unresectable CRLM, a group previously resigned to dismal outcomes with palliative chemotherapy alone. When compared to LT for HCC within Milan criteria, recurrence of malignancy remains more common after LT for CRLM, though these recurrences are generally responsive to treatment and many patients experience prolonged disease-free survival even after detection of recurrence. The significant advances in five-year overall survival seen between SECA I and the more modern trials of this decade suggest that success after LT for CRLM should be measured by delaying the onset of recurrence after transplant and prolonging post-recurrence survival rather than preventing recurrence completely. Further research will help improve our understanding of each patient’s unique tumor molecular and biological activity prior to transplant and how it can be used to better optimize treatment and risk-stratify them prior to transplant. New and advanced surgical approaches and the expanded use of LDLT will also help expand the cohort of patients who can undergo LT for CRLM, which will both vastly improve survival for CRLM but also create new and unique challenges in the field of transplant oncology.

## Figures and Tables

**Table 1:** Recurrence and overall survival after liver transplant for isolated colorectal liver metastasis.

Study	Year	N	Median F/U, Months (Range)	Estimated OS	Recurrence Rate (%)	One-Year DFS (%)	Site of Recurrence ^1^ (%)	
Hagness et al. [8]	2013	21	27 (8–60)	60% at 5 years	90	35	Lung—19 (100.0) Liver—7 (36.8) Bones—5 (26.3)	Ovaries/Adrenals—3 (15.8) Peritoneum—1 (5.3) Lymph Nodes—2 (10.5)
Dueland et al. [13]	2020	15	36 (5–60)	83% at 5 years	53	53	Lung—6 (75.0)	Other—2 (25.0)
Smedman et al. [16]	2020	10	23 (6–26)	43% at 2 years	80	30	Lung—6 (75.0) Liver—1 (12.5) Pelvis—1 (12.5)	Peritoneum—1 (12.5) Lymph Nodes—1 (12.5)
Hernandez-Alejandro et al. [14]	2022	10	18 (4.8–34.8)	100% at 1.5 years	30	62	Liver—1 (33.3) Other–1 (33.3)	Peritoneum—1 (33.3)
Rajendran et al. [18]	2023	7	15 (13.5–30.2)	100% at 3 years	29	86	Lung—1 (50.0)	Lymph Nodes—1 (50.0)
Sasaki et al. [19]	2023	46	12 (6.1–22.4) ^2^	60% at 3 years	22	75	NR	
Byrne et al. [20]	2024	20	NR	90% at 3 years	NR	90	Lung—4 Liver—1	Peritoneum—2
Adam et al. [17]	2024	38	59 (42.4–60.2) ^2^	73% at 5 years	72	83	Liver—1 (4.0) Lungs—14 (54.0) Other—5 (19.0)	Lymph Nodes—3 (12.0) Multiple—3 (12.0)
Wehrle et al. [21]	2025	26	21 (11–36) ^2^	85% at 2 years	31	83	Lung—3 (37.5) Liver—2 (25.0)	Colorectal—1 (12.5) Multiple—2 (25.0)

F/U = Follow-up. OS = Overall survival. DFS = Disease-free survival. NR = Not reported. ^1^ For patients with multiple sites of recurrence, some studies listed each location separately while others reported them as “multiple”. We present the sites of recurrence here in the manner originally reported in each study. ^2^ Reflects the interquartile range (IQR) of follow-up as reported in the original article rather than complete range of follow-up, which was not reported.

**Table 2:** The Fong clinical risk score.

Fong Clinical Risk Score (0–5)	
1	Lymph node-positive primary tumor
2	Interval from primary colorectal tumor detection to metastasis < 12 months
3	>1 Hepatic tumor
4	Largest hepatic tumor diameter > 5 cm
5	Plasma carcinoembryonic antigen level > 200 ng/mL

**Table 3:** The Oslo score.

Oslo Score (0–4)	
1	Largest hepatic tumor diameter > 5.5 cm
2	Plasma carcinoembryonic antigen level > 80 ng/mL
3	Interval between primary colorectal tumor resection and liver transplant < 2 years
4	Progressive disease despite systemic chemotherapy at the time of liver transplant

## Data Availability

No new data were created or analyzed in this study. Data sharing is not applicable to this article.

## Abbreviations

The following abbreviations are used in this manuscript:

LT	Liver transplant
CRC	Colorectal cancer
CRLM	Colorectal liver metastases
HCC	Hepatocellular carcinoma
LDLT	Living-donor liver transplant
DFS	Disease-free survival
ctDNA	Circulating tumor DNA
MTV	Metabolic tumor volume
FCRS	Fong clinical risk score
CEA	Carcinoembryonic antigen
